# Unraveling the Effects of Reducing and Oxidizing Pretreatments and Humidity on the Surface Chemistry of the Ru/CeO_2_ Catalyst during Propane Oxidation

**DOI:** 10.1021/acs.jpcc.4c08033

**Published:** 2025-01-14

**Authors:** Thu Ngan Dinhová, Oleksii Bezkrovnyi, Lesia Piliai, Ivan Khalakhan, Samiran Chakraborty, Maciej Ptak, Piotr Kraszkiewicz, Mykhailo Vaidulych, Michal Mazur, Štefan Vajda, Leszek Kepinski, Michael Vorochta, Iva Matolínová

**Affiliations:** 1 Department of Surface and Plasma Science, Faculty of Mathematics and Physics, Charles University, V Holešovičkách 2, Prague 180 00, Czechia; 2 W. Trzebiatowski Institute of Low Temperature and Structure Research, Polish Academy of Sciences, Okólna 2, Wroclaw 50-422, Poland; 3 Department of Nanocatalysis, J. Heyrovský Institute of Physical Chemistry, Czech Academy of Sciences, Dolejškova 2155/3, Prague 182 23, Czechia; 4 Department of Physical and Macromolecular Chemistry, Faculty of Science, Charles University, Hlavova 8, Prague 128 43, Czechia

## Abstract

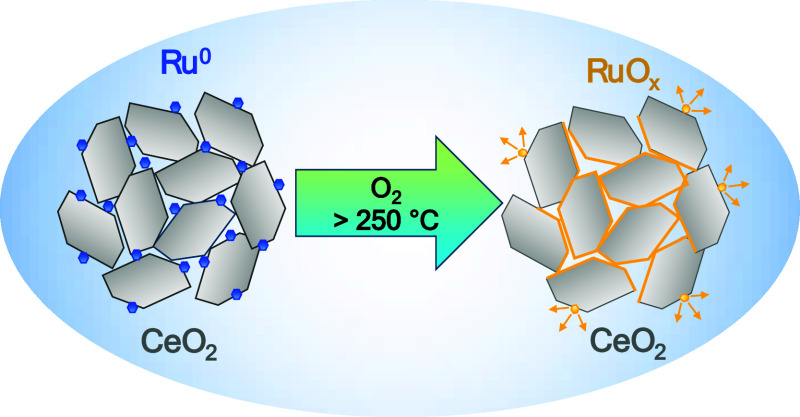

This work investigates the surface chemistry of the Ru/CeO_2_ catalyst under varying pretreatment conditions and during the oxidation of propane, focusing on both dry and humid environments. Our results show that the Ru/CeO_2_ catalyst calcined in O_2_ at 500 °C initiates propane oxidation at 200 °C, achieves high conversion rates above 400 °C, and demonstrates almost no change in activity in the presence of water vapor across the entire studied temperature range of 200–500 °C. Prereduction of the oxidized Ru/CeO_2_ catalyst in H_2_ significantly enhances its activity, though this enhancement diminishes at higher temperatures. Adding water to the reaction mixture boosts the low-temperature activity of the prereduced catalyst but decreases it at 300–400 °C. Several *ex-situ* analytical techniques in combination with the *in-situ* NAP-XPS analysis reveal that while exposed to oxygen, Ru nanoparticles on the ceria surface oxidize to form RuO_2_ below 200 °C and volatile RuO_*x*_ (*x* > 2) at higher temperatures. The presence of water vapor in the reaction mixture leads to the transformation of RuO_2_ into ruthenium hydroxide at 200 °C, which, in turn, facilitates propane oxidation. At higher temperatures, the water does not have much influence on the oxidation state of Ru but slightly inhibits its evaporation from the surface. It is also demonstrated that Ru in the Ru/CeO_2_ catalyst exists predominantly in the Ru^n+^ (*n* > 4) oxidation states at typical VOC oxidation temperatures rather than the expected Ru^4+^ state.

## Introduction

Ceria-supported platinum-group metal (PGM) nanoparticles (NPs) are widely used catalysts for the total oxidation of volatile organic compounds (VOC), which may cause numerous health problems to humans. Their catalytic activity is mainly determined by two factors. The first one is the reversible redox Ce^4+^/Ce^3+^ transition in ceria, thanks to which ceria functions as an oxygen reservoir in a process that determines the ability of ceria-based catalysts to supply oxygen to the active sites via the Mars-van Krevelen (MvK) reaction mechanism.^1,2^ The second factor is the ability of PGMs to cleave C–C and C–H bonds, an essential step in complete VOC oxidation.^3,4^ Among various PGMs, Ru has attracted particular attention as an active metal component in different catalyst formulas for VOC oxidation due to its high activity and relatively low price.^5,6^

Although the catalytic activity of Ru/CeO_2_-based materials has been extensively studied for decades,^7−9^ the question of the nature of active sites in VOC oxidation under realistic conditions remains open. The major challenge in elucidating this problem is the wide range of the possible Ru oxidation states (form −2 to +8) reported in the literature.^10,11^ The most common Ru oxidation states detected in Ru/CeO_2_ catalysts are Ru^0^ and Ru^4+^. Reduction in H_2_, a standard procedure for activating Ru-based catalysts,^10,12^ suggests that Ru is expected to be in the metallic form before the reaction. However, while discussing the nature of active sites in Ru-based catalysts, the problem of the redox process occurring in these catalysts under working conditions is rarely addressed at its full complexity. Moreover, the reaction of VOC oxidation typically occurs in the presence of H_2_O vapor that usually harms M/CeO_2_ catalysts due to the strong adsorption of H_2_O molecules on the abundant oxygen vacancies of CeO_2_ support that hinders the adsorption and activation of both C_3_H_8_ and O_2_.^13^ On the other hand, a recent study by Wang et al., reported a significant increase in the activity of the Ru/CeO_2_ catalyst during propane oxidation at 210 °C in the presence of water vapor, which was attributed to the redispersion of Ru under hydrothermal conditions.^14^ It is also reported that OH groups on the ceria surface may help stabilize Ru–O_*x*_ complexes.^15^

Since the typical working temperatures during the VOC oxidation (150–400 °C) can introduce reversible chemical changes in Ru-based catalysts on a broad scale,^16^*in-situ* characterization of this catalyst under working conditions is indispensable. Near-ambient pressure X-ray photoelectron spectroscopy (NAP-XPS) is a powerful technique that enables the straightforward analysis of the catalyst’s chemical state under gas exposure at mbar-range pressures and high temperatures.^17^ Despite operating at 2 orders of magnitude lower pressures, NAP-XPS can provide useful approximations and valuable insights into chemical reactions that proceed at ambient pressures.^18^ Our previous work studying Ru NPs supported on polycrystalline hollow nanospheres of ceria confirmed the importance of using NAP-XPS to study the Ru/CeO_2_ interaction with C_3_H_8_+O_2_ gas medium (C_3_H_8_ was chosen as a model VOC).^19^ The study revealed the formation of a volatile Ru^n+^ (*n* > 4) oxide. Exposing the catalyst to air and cooling to room temperature (RT) resulted in fast Ru^n+^ reduction to Ru^4+^, making its detection impossible with *ex-situ* techniques. The study also identified a possible reason for the Ru/CeO_2_ catalyst deactivation during operation: evaporation of the volatile RuO_3_/RuO_4_.

In this work, we performed a comprehensive comparative investigation of the stability and activity of Ru supported by polycrystalline CeO_2_ during propane oxidation under dry and humid conditions. The study includes the measurements of the catalyst performance in a bed reactor and its characterization using different *ex-situ* experimental techniques, such as the temperature-programmed reduction in hydrogen (H_2_-TPR), X-ray diffraction (XRD), transmission electron microscopy (TEM), X-ray photoelectron spectroscopy (XPS), and Raman spectroscopy. Another substantial part of the article presents the *in-situ* NAP-XPS study. The obtained results allowed us to determine the effect of water on the stability and activity of the Ru/CeO_2_ catalyst in propane oxidation. This information is essential for understanding the catalyst behavior under the industrially relevant conditions of VOC oxidation.

## Experimental Section

### Catalyst Synthesis

CeO_2_ support was synthesized by annealing Ce(NO_3_)_3_·6H_2_O precursor at 500 °C for 3 h in the air. The Ru/CeO_2_ catalyst, with a Ru loading of 2 wt %, was prepared using the deposition–precipitation method. Initially, 1.0 g of CeO_2_ NPs were suspended in 40 mL of water, followed by adding the desired amount of an 11 wt % solution of Ru in HNO_3_. The suspension pH was adjusted to 9.5 using an aqueous ammonia solution, and then the resulting suspension was aged and stirred at 25 °C for 15 min. The precipitate was separated by centrifugation, thoroughly washed with distilled water, and dried overnight at 60 °C. Subsequently, the resulting powder was annealed in a flow of 5% H_2_ diluted in He at 500 °C for 180 min. In the text, such a prepared catalyst is called “as-prepared” Ru/CeO_2_.

### Characterization Techniques

Transmission electron microscopy (TEM) characterization was done using a JEOL NEOARM 200 F microscope equipped with a spherical aberration corrector, a Schottky-type field emission gun operating at an accelerating voltage of 200 kV, and a high-angle annular dark field (HAADF) detector. Studied samples were wiped onto a holey carbon-coated copper grid and characterized in scanning mode HAADF-STEM. Corresponding energy dispersive spectroscopic (EDS) maps were acquired using a JEOL JED-2300 detector.

The temperature-programmed reduction in hydrogen (H_2_-TPR) experiment was performed with an Autochem II 2920 instrument (Micromeritics). Hydrogen consumption was measured three times for each catalyst, between −50 and 500 °C (ramp rate -10 °C/min) in a flow of 5% H_2_/Ar (30 cm^3^/min). All used gases were of high purity (at least 99.999%). Typically, 50 mg of the sample were placed in a quartz-glass reactor and heated in helium (30 cm^3^/min) at 150 °C for 10 min to clean the catalyst surface. Then, it was cooled down in helium to −50 °C, and the first H_2_-TPR measurement (TPR#1) was performed. After that, the sample was cooled to RT in a hydrogen atmosphere, flushed with helium for 15 min, and reoxidized by heating in synthetic air at 500 °C for 60 min (10 °C/min). The reoxidized sample was cooled in synthetic air to RT, flushed with helium for 15 min, and cooled to −50 °C in helium. Then, helium was switched to hydrogen, and the second H_2_-TPR measurement (TPR#2) was performed. In the third H_2_-TPR measurement (TPR#3), the sample was treated according to the procedure described for TPR#2.

Raman spectra in the 125–1300 cm^–1^ range were collected using a Renishaw InVia Raman spectrometer equipped with a confocal optical microscope. An argon laser operating at 514.5 nm was used as an excitation source. The laser spot was approximately 1.5 mm in diameter. Each spectrum was collected three times with a 25-s acquisition time.

*Ex-situ* XPS and NAP-XPS measurements were performed in a laboratory system provided by SPECS Surface Nano Analysis GmbH, equipped with a monochromated high-intensity Al Kα X-ray source (1486.6 eV) with a beam spot size of 0.5 mm, a high-pressure (NAP) cell, and a multichannel electron energy analyzer (Specs Phoibos 150) coupled with a differentially pumped electrostatic prelens system. About 5 mg of Ru/CeO_2_ powder was pressed into a fine 5 × 5 mm^2^ tungsten mesh using a hydraulic press at 80 kN/m^2^ pressure, spot-welded to a sample holder, and loaded into the NAP cell. It was possible to investigate the sample inside the cell in an ultrahigh vacuum (UHV) or during exposure to various gaseous atmospheres in the mbar range at temperatures ranging from 25 to 450 °C. The measurements under dry conditions were conducted in a 1.8 mbar O_2_:C_3_H_8_ (10:1) mixture, while humid conditions involved introducing an additional 1 mbar of water vapor to create O_2_:C_3_H_8_:H_2_O mixtures with total pressures of 2.8 mbar, respectively. During the exposures, Ce 3d, O 1s, C 1s, and Ru 3d XPS spectra were acquired at the analyzer pass energy of 20 eV. The obtained spectra were processed using KolXPD software.

### Catalytic Activity Tests

The catalytic activity of the Ru/CeO_2_ catalyst for propane oxidation under dry and humid conditions was studied using a microcapillary reference reactor equipped with quartz capillary tube (outer diameter −1 mm, thickness −0.4 mm, and length −50 mm) and two 30 mm long ceramic tubes wrapped with tungsten wire as heating elements placed alongside the capillary. The temperature of the reaction was controlled by a combination of an electronic temperature controller (Eurotherm 2404), a power supply (Kepco), and an in-bed thermocouple (type K, Omega). The reactant gases were introduced using a gas mixer (Swagelok) equipped with the assembly of mass-flow controllers (Brooks). The pressure inside the reactor was maintained at 800 Torr, which was achieved by implementing a regulation loop employing a downstream mass-flow controller (Brooks) connected to a diaphragm pump (Divac 1.4HV3, Pfeiffer) and a pressure transducer (PX209, Omega). The readout from a pressure transducer was monitored by custom software written in Python, adjusting the flow through a downstream controller as needed to maintain the preset pressure.

For each performed catalyst activity testing, ∼ 0.8 mg of the as-prepared Ru/CeO_2_ powder was loaded into the capillary and fixed with quartz wool on both sides. Prior to the measurements, the reactor was evacuated using a membrane pump and flushed with helium flow. Next, all catalysts were treated in 10% O_2_/He at 450 °C for 150 min. The catalytic measurements were then performed at a pressure of 1.05 bar without and with added water vapor into the feed, i.e., under dry and humid conditions, respectively. Under dry conditions, the C_3_H_8_ to O_2_ ratio was 1:10, with concentrations of 800 and 8000 ppm, respectively, balanced in helium. Under humid conditions, the ratio of reactants was kept unchanged as under dry conditions, but with 400 ppm of H_2_O/He used to balance, resulting in a water concentration of 358 ppm. It was the maximum water concentration allowed by the mass-flow controllers to supply gases to the catalytic reactor. The used concentrations of the reactants corresponded to the partial pressures of 8, 0.8, and about 0.04 mbar O_2_, C_3_H_8_ and H_2_O, respectively, and were in the same pressure order as those used in the NAP-XPS studies. The total gas flow in all catalytic tests was kept constant at 20 mL/min. The catalytic oxidation of propane on different samples was recorded by gradually increasing the temperature from room temperature (RT) to 500 °C followed by a subsequent decrease back to RT with a 100 °C step (ramp rate −5 °C/min) (following the ramp outlined in Figure S1 of the Supporting Information (SI)). The identity and concentrations of the reaction products were determined using gas chromatography (GC, Inficon MicroGC Fusion). GC was equipped with Rt-Molsieve 5A (0.25 mm ID 10 m), Rt-Q Bond (0.25 mm ID, 12 m), and Rxi-1 ms (0.15 mm ID, 20 m) columns and thermal conductivity detectors (TCD). The retention times and response factors were determined from C_3_H_8_ (3% in He, Linde) and CO_2_ (10% in He, Linde) standards. The CO_2_ formation rate (R_CO2_) is presented as the molar rate per gram of a catalyst (Ru/CeO_2_) per second (mol g_cat_^–1^ s^–1^), while the total propane conversion rate (R_C3H8_) is presented as the molar rate per gram of used Ru per second (mol g_Ru_^–1^ s^–1^) to enable comparison with the literature data. Under high-temperature conditions, a tiny amount of propene (<2%) was also detected as a product of oxidative dehydrogenation. Importantly, the empty capillary reactor, with quartz wool and without a catalyst, showed no catalytic propane conversion under any of the applied test reaction conditions.

## Results and Discussion

### Catalyst Morphology and Structure

**Figure 1:**
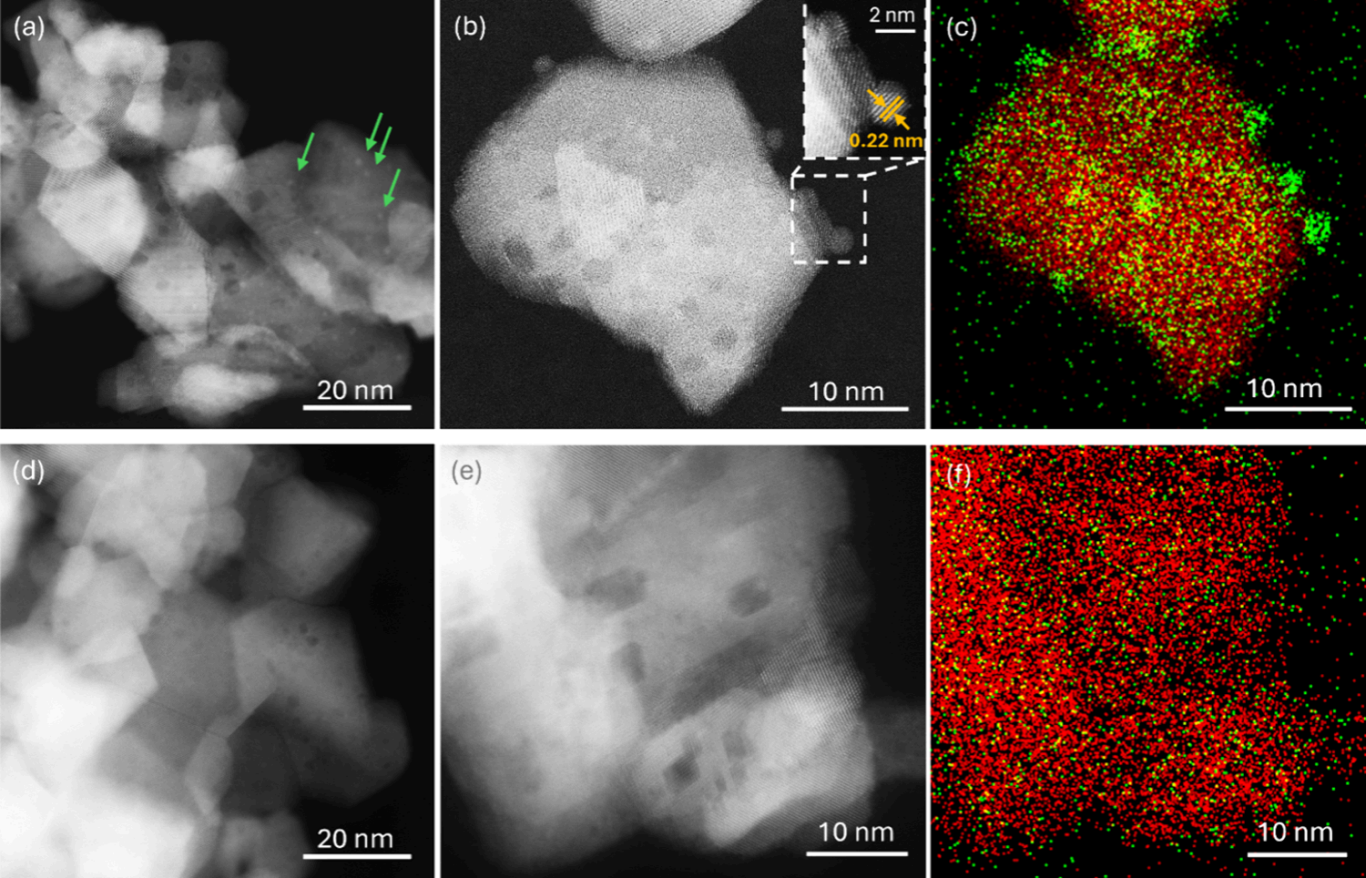
HAAD-STEM images and the corresponding STEM/EDS elemental distribution maps of Ru (green) and Ce (red) distributions for the as-prepared (a, b, c) and O_2_-calcined (d, e, f) Ru/CeO_2_ catalysts.

A combination of HAADF-STEM and STEM/EDS analyses was used to study the structure and morphology of the as-prepared (reduced) and calcined in 10% O_2_/He at 450 °C catalysts at the micro level. The HAAD-STEM images and the corresponding EDS maps are presented in Figure 1. The images of the as-prepared Ru/CeO_2_ powder revealed small NPs with a lattice spacing of 0.22 nm, supported by large crystallites with characteristic lattice spacings of 0.31 and 0.27 nm. The lattice spacing for small NPs can be associated with the (200) lattice planes of RuO_2_,^20^ while those in the large crystallites correspond to the (111) and (200) lattice planes of CeO_2_, respectively.^21−23^ These results agreed with the XRD pattern (Figure S2 of the SI), exhibiting only characteristic diffraction peaks of cubic fluorite CeO_2_ with a mean crystallite size of approximately 30 nm. No RuO_2_ reflections were detected in the XRD diffractogram, likely due to the small size and low concentration of RuO_2_ NPs. In contrast, the images of the oxidized catalyst revealed only CeO_2_ crystallites with no Ru nanoparticles visible. The EDS map in Figure 1f confirmed a homogeneous distribution of Ru across the ceria surface. Additionally, the STEM/EDS spectra corresponding to maps in Figures 1c and 1f indicated a slight decrease in Ru concentration, from approximately 5% to 3% (see Figure S3 of the SI).

### H_2_-TPR Characterization

**Figure 2:**
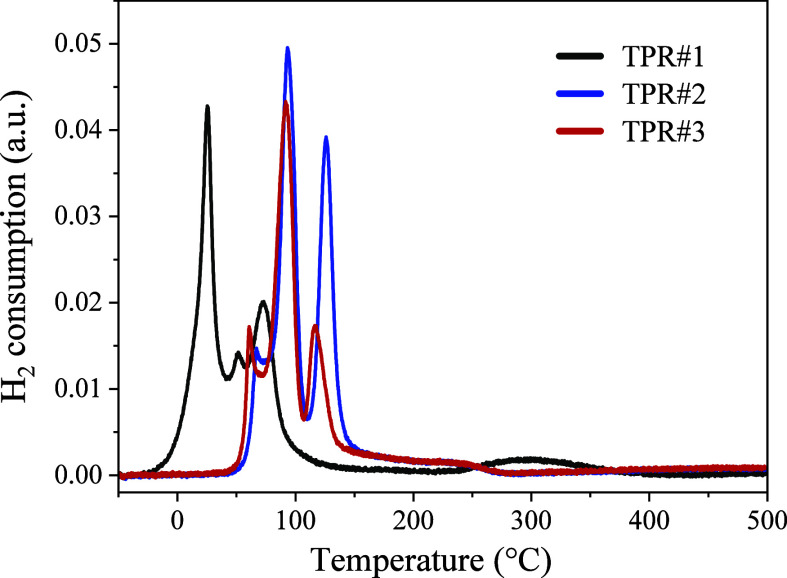
Temperature-programmed reduction profiles for the as-prepared Ru/CeO_2_ catalyst.

The reducibility of the as-prepared Ru/CeO_2_ catalyst was investigated by H_2_-TPR. Considering the number of possible processes that could occur in the Ru/CeO_2_ catalyst under its alternating oxidizing/reducing treatment (e.g., possible changes of the chemical state of Ru or its diffusion into ceria), three subsequent H_2_-TPR cycles (TPR#1–3) were performed in a temperature region from −50 to 500 °C with the reoxidation in synthetic air at 500 °C between each cycle. The obtained H_2_-TPR profiles are presented in Figure 2.

Even though the as-prepared Ru/CeO_2_ catalyst was reduced during the synthesis, the TPR#1 test showed its reduction already at a temperature below 0 °C, forming an intense reduction peak centered around 25–30 °C. In addition, two smaller reduction peaks were recorded at about 50 and 75 °C, together with a tiny broad peak between 250 and 400 °C. The significant amount of hydrogen consumed during TPR#1 suggests that the exposure of the initially reduced as-prepared catalyst to ambient air reoxidizes it, providing oxygen species that can interact with hydrogen already at RT. On the other hand, the *in situ* reoxidation of the catalyst in synthetic air at 500 °C completely changed its reduction properties. Similar to TPR#1, the following TPR#2 profile revealed three well-distinguishable peaks, however, different in both intensity and temperatures at which they appeared. A small and narrow peak was detected at about 70 °C, and two more intense peaks were seen at about 95 and 125 °C. The last peak exhibited tailing extending to about 275 °C. The TPR#3 profile recorded after the second catalyst reoxidation was similar to the TPR#2 cycle. However, all measured reduction peaks showed a slight shift toward lower temperatures (about 10–15 °C), and the peak at 115 °C was approximately two times smaller than the corresponding peak measured during TPR#2. Quantitative analysis of the H_2_-TPR results showed that the amount of H_2_ used to reduce 50 mg of the as-prepared catalyst during TPR#1 was 27.8 μmol. It should be mentioned that the calculated amount of H_2_ required to reduce RuO_2_ is 19.8 μmol (assuming 2 wt % Ru content). Heating the sample in synthetic air at 500 °C slightly increased the amount of H_2_ consumed during TPR#2 to 30.4 μmol, while the total amount of H_2_ consumed during TPR#3 was 26.2 μmol.

It should be mentioned that for stoichiometric cerium oxide NPs, the surface reduction of Ce^4+^ to Ce^3+^ usually occurs between 300 and 500 °C.^24−26^ In the case of RuO_2_, the reduction at temperatures starting from 100 °C has been reported.^27−29^ On the other hand, the reoxidation of reduced ceria at low temperatures typically leads to the formation of various weakly bonded superoxide and peroxide (O_2_^–^ and O_2_^2–^) species on the surface.^30,31^ Thus, we assume that the peaks at 25 and 50 °C in TPR#1 may arise from the H_2_ interaction with these species adsorbed on the ceria surface. It is reported that such interaction at low temperatures leads not to water formation but to hydroxylation of the ceria surface by OH groups.^15^ On the other hand, as detailed below, no superoxide or peroxide species were identified in the Raman spectra. An alternative explanation for the observed peaks at 25 and 50 °C could be the incorporation of H_2_ into the reduced ceria, as reported in the study by Z. Li et al.^32^ The peak at about 75 °C most probably originated from the reduction of the RuO_2_ NPs. Their small (2–3 nm) sizes might explain a slightly lower reduction temperature compared to the literature value. Finally, as the ceria surface was completely reduced before exposure to air, covering it with the weakly bonded oxygen species resulted only in a very weak peak corresponding to the surface oxygen reduction at about 300 °C.

The reoxidation of the catalyst in synthetic air at 500 °C before TPR#2 and TPR#3 cycles resulted in the complete reoxidation of ceria and, as will be shown below, in the oxidation of the Ru NPs into volatile RuO_*x*_ (*x* > 2) species, redispersing in a thin RuO_*x*_ layer on the surface of ceria crystallites. This process likely includes the formation of stronger Ru–O–Ce bonds at the catalyst surface, requiring a higher reduction temperature of about 100 °C compared to the RuO_2_ NPs. In addition, the appearance of metallic Ru NPs at 100 °C, capable of dissociating H_2_, facilitated the subsequent substantial reduction of the CeO_2_ NPs already at 130–150 °C. It is also plausible that there is a continuous loss of ruthenium due to the flushing of volatile RuO_*x*_ species from the reactor, explaining the observed decrease in H_2_ consumption between the TPR#2 and TPR#3 reduction cycles.

### Catalytic Properties

The time- and temperature-dependent catalytic activity tests of differently pretreated (oxidized and reduced) Ru/CeO_2_ catalysts for propane oxidation were assessed under dry (0.08 vol % C_3_H_8_–0.8 vol % O_2_–99.12 vol % He) and humid (0.08 vol % C_3_H_8_–0.8 vol % O_2_–0.04% H_2_O–99.08 vol % He) conditions. To obtain the oxidized catalyst, the as-prepared Ru/CeO_2_ powder underwent annealing in a 10% O_2_ in He flow at 450 °C for 150 min inside the catalytic reactor. The reduced Ru/CeO_2_ catalyst was obtained through annealing inside the reactor in a 10% H_2_ in He atmosphere at 450 °C for 150 min. An additional H_2_ reduction was required due to the reoxidation of the as-prepared catalyst in atmospheric air, as revealed by TEM and H_2_-TPR studies.

**Figure 3:**
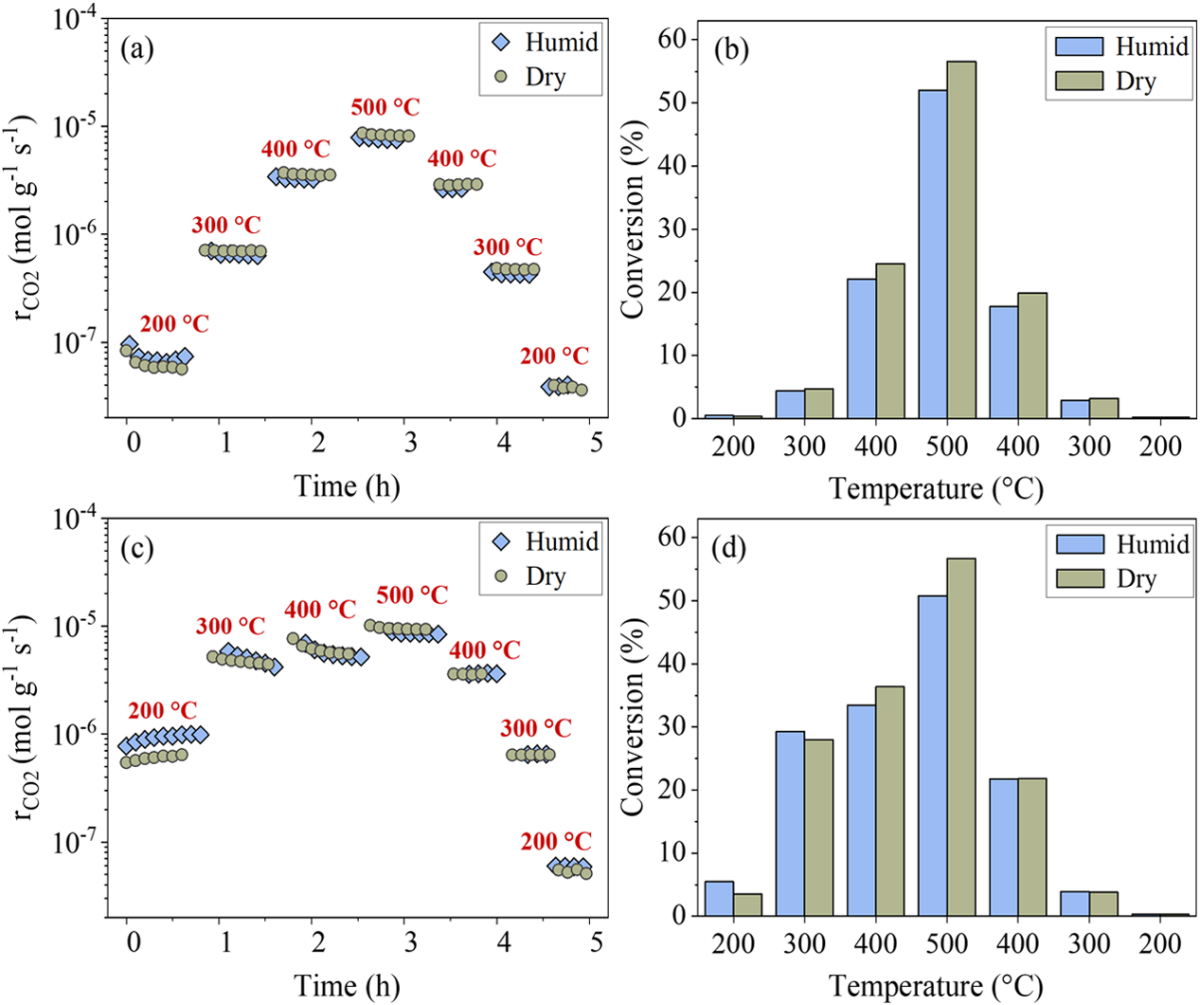
Time- and temperature-dependent CO_2_ production rates and the total propane conversion during the catalytic oxidation of propane over the oxidized (a, b) and reduced (c, d) Ru/CeO_2_ catalysts under dry and humid conditions.

Figures 3a, b illustrate the time-resolved CO_2_ production rates and the average propane conversions obtained during propane oxidation over the oxidized Ru/CeO_2_ catalyst while increasing temperature from 200 to 500 °C and decreasing it back to 200 °C under both dry and humid conditions. Table S1 in the SI also provides the reaction rates (R_C3H8_, mol g_Ru_^–1^·s^–1^), which are compared with the literature data to highlight the catalyst’s performance and quality. It should be noted that besides CO_2_, a small amount of propene (<2%) was also detected as the product of propane oxidation. Under dry conditions, the oxidized catalyst exhibited high and stable catalytic propane conversion, increasing with temperature and reaching approximately 58% conversion at 500 °C. The catalyst’s activity was somewhat lower during the decreasing temperature ramp. This suggested the presence of a process responsible for catalyst deactivation during propane oxidation at high temperatures. Under humid conditions, the oxidized catalyst exhibited almost identical behavior, showing very similar catalytic activity and the same deactivation after annealing at 500 °C.

In order to assess the reaction order with respect to the pressure of propane supplied to the reactor, we also conducted propane oxidation tests in dry conditions with a flow stoichiometry of 0.24 vol % C_3_H_8_, 2.4 vol % O_2_, and 97.36 vol % He, i.e., 3 times higher propane concentration (see Figure S4 of the SI). As illustrated in Figure S5 of the SI, the results demonstrated a linear dependence of the CO_2_ production rate on the propane pressure, indicating a first-order reaction with respect to propane pressure. The activation energy *E*_*a*_ estimated for propane oxidation reaction under dry conditions over the oxidized catalyst was found to be approximately 52 kJ/mol (Figure S5b of the SI). This value is lower than the activation energies reported in the literature for propane oxidation over polycrystalline Ru/CeO_2_ catalysts with similar Ru loadings (1–3 wt %), which typically range from 58.6 to 72.1 kJ/mol.^12^ However, it remains higher than the activation energy of 28.6 kJ/mol observed on a catalyst containing 2 wt % Ru supported on CeO_2_ nanorods.^33^

The reaction rates observed on the reduced Ru/CeO_2_ catalyst are presented in Figures 3c, d and Table S1 in the SI. It can be seen that the reduction of Ru/CeO_2_ substantially increases its catalytic activity toward propane oxidation at lower temperatures up to 400 °C on the rising branch of the temperature ramp. The *E*_*a*_ for the reduced catalyst in dry conditions decreased to 27 kJ/mol (Figure S5b of the SI), a value comparable to that previously reported for Ru supported on CeO_2_ nanorods. At 200 °C, the propane conversion under dry conditions was approximately 1 order of magnitude higher than in the case of the oxidized catalyst, followed by a relative increase of approximately 60% and 70% at 300 and 400 °C, respectively. The slight activation observed for the reduced catalyst at 200 °C is likely related to carbon contamination, which, as evidenced by NAP-XPS measurements presented below, remains on the catalyst surface after reduction in H_2_. Under reaction conditions, this contamination likely oxidizes, freeing additional adsorption sites and thereby enhancing reactivity. Above 300 °C, a slight catalyst deactivation was observed at each temperature step on the temperature-increasing branch of the applied temperature ramp, and the impact of the reduction began to diminish and completely vanish at 500 °C. At this temperature, the reduced catalyst exhibited very similar conversion rates to the ones obtained for the oxidized catalyst, which may indicate that the nature of the oxidized and reduced catalysts converges when reaching 500 °C. This hypothesis is further confirmed by the comparable rates observed for the oxidized and reduced catalysts on the temperature-decreasing branch of the temperature ramp. As discussed in the NAP-XPS section, we relate this deactivation to the oxidation of Ru NPs and the partial washing out of volatile RuO_*x*_ species from the catalyst.

The presence of water vapor in the reaction mixture substantially affected the catalytic activity of the reduced Ru/CeO_2_ catalyst only at 200 °C, resulting in a relative increase of 55% in propane conversion, from 3.5% to 5.4%. A similar effect of a significant increase in Ru/CeO_2_ catalyst activity during propane oxidation at 210 °C was also reported by Wang et al., who conducted the measurements in the presence of 5% water at atmospheric pressure.^14^ Starting from 300 °C, the conversions measured under dry and humid conditions differed only slightly. It should also be noted that both dry and humid measurements on the reduced catalyst, conducted at 200–300 °C, demonstrated complete propane oxidation. However, at 400–500 °C, a small amount (up to ca. 2%) of propene was detected at the reactor outlet, a pattern similar to that observed with the oxidized catalyst.

### *Ex-Situ* Raman and XPS Characterizations

**Figure 4:**
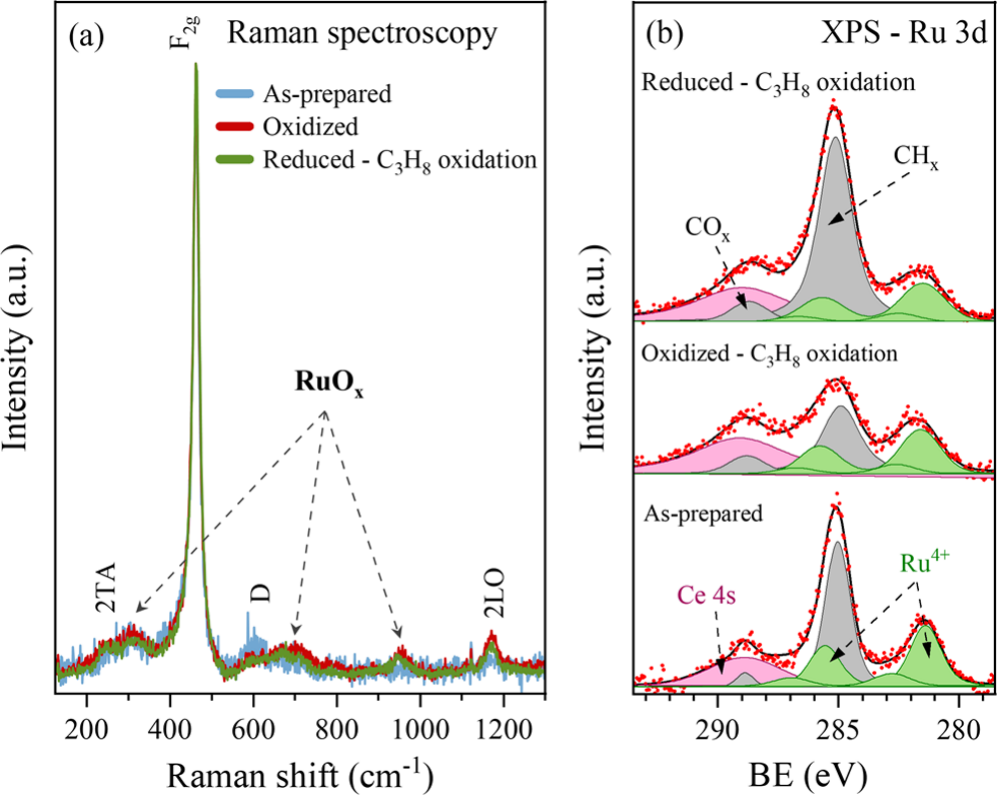
(a) *Ex-situ* measured Raman spectra of the as-prepared and oxidized Ru/CeO_2_ catalysts and the reduced Ru/CeO_2_ catalyst tested for propane oxidation under dry conditions; (b) *ex-situ* measured XPS Ru 3d spectra of the as-prepared Ru/CeO_2_ catalyst, and the oxidized and reduced Ru/CeO_2_ catalysts tested for propane oxidation under dry conditions.

*Ex-situ* Raman and XPS analyses were further performed to investigate the influence of different reaction environments on the chemical state of the as-prepared Ru/CeO_2_ catalyst. Figure 4a shows the background-subtracted Raman spectra collected from the as-prepared Ru/CeO_2_ catalyst, the catalyst oxidized in O_2_ at 500 °C, and the reduced catalyst tested for propane oxidation under dry conditions. It can be seen that all three spectra were dominated by strong Raman bands at 463 cm^–1^ originating from CeO_2_ (F_2g_).^34^ Weak bands at 248, 593, and 1171 cm^–1^ are related to second-order transverse acoustic (2TA), the ceria defect (D), and second-order longitudinal optical (2LO) modes, respectively.^21^ The remaining bands observed at 316, 667, 693, 711, and 955 cm^–1^ can be assigned to various forms of Ru.^10,35^ It can be seen that the as-prepared sample revealed a more intense D-band, indicating a more defective ceria structure containing Ce^3+^ ions. Apart from being due to ceria defects, some contributions to the band at ∼590 cm^–1^ may originate from hydrated RuO_2_,^36^ as supported by XPS data (see below). At the same time, the Raman spectra of the samples exposed to the oxidizing atmospheres were similar, with weak bands observed between 667 and 711 cm^–1^ and a band at about 955 cm^–1^, indicating the presence of Ru^4+^ ions in the CeO_2_ structure, Ru–O–Ce groups, or RuO_2_.^37,38^ The lower intensity of these bands in the spectrum of the as-prepared catalyst, which is expected to contain RuO_2_ species, may indicate that they likely emerge only after the high-temperature oxidation of Ru NPs. This process leads to the fine dispersion of Ru on the ceria surface and the formation of robust Ru–O–Ce bonds, as will be discussed below.

We also focused on the spectral ranges of 1050–1150 cm^–1^ and 850–950 cm^–1^. For nanocrystalline ceria, the Raman vibration frequencies for O_2_^–^ and O_2_^2–^ species are expected to fall within these ranges.^30,39,40^ Such species could potentially explain the low-temperature peaks observed in the H_2_-TPR spectrum of the as-prepared catalyst. Additionally, they may play a role in propane oxidation at low temperatures, contributing to the enhanced activity of the reduced Ru/CeO_2_ catalyst at 200 °C. However, these spectral regions did not exhibit clear bands. Instead, they coincided with weak shoulders attributable to RuO_x_ species, with a maximum at approximately 950 cm^–1^, or with the 2LO band near 1170 cm^–1^. It is also plausible that the concentrations of these oxygen species are below the detection threshold, or that the Raman measurements conducted using a confocal microscope are less sensitive due to the limited sampling area.

Figure 4b presents the Ru 3d XPS spectra acquired in UHV from the as-prepared Ru/CeO_2_ catalyst and differently pretreated Ru/CeO_2_ catalysts tested in the catalytic reactor for propane oxidation under dry conditions. Similarly, as in the case of the TEM analysis, Ru in the as-prepared Ru/CeO_2_ sample was detected only in the Ru^4+^ form that can be identified from the Ru 3d doublet with the main peak positioned at binding energy (BE) of 281.3 eV and the spin–orbital split of about 4.2 eV.^12,41^ The doublet was at a higher BE than the typically reported value of 281 eV for anhydrous RuO_2_, indicating the presence of hydrous RuO_2_ in the catalyst.^42^ Small satellite peaks accompany this doublet at approximately 1.5 eV higher BE. These findings corroborate the TEM and Raman results, which demonstrate that the tiny (2–3 nm) Ru NPs undergo complete oxidation in ambient atmosphere even at room temperature. The surface of the as-prepared catalyst also exhibited a relatively high amount of carbon contamination, as evident from the C 1s signal, which overlaps with the Ru 3d and Ce 4s signals. A strong peak at about 285 eV was assigned to different carbonaceous species containing C–C and C–H_*x*_ bonds.^43,44^ In contrast, the smaller peak at about 289.5 eV probably originated from various carbonate and carboxylate species.^43^ The catalysts that underwent oxidizing or reducing pretreatments, followed by propane oxidation tests in the catalytic reactor, exhibited almost identical spectra as for the as-prepared catalyst. The only difference was in the amount of carbon contamination. The oxidized catalyst showed less, and the reduced one exhibited more carbon on the surface than the as-prepared catalyst.

The Ce 3d and O 1s spectra measured on the as-prepared Ru/CeO_2_ catalyst and on the oxidized and reduced Ru/CeO_2_ catalysts after the test in the catalytic reactor for propane oxidation are shown in Figure S6 in the SI. It can be seen that the Ce 3d spectra looked the same for all three samples and consisted of three spin–orbit split doublets of CeO_2_.^45−47^ The O 1s spectra contained two peaks at about 529.5 and 531.5 eV. The first peak typically belongs to lattice oxygen in CeO_2_,^45,47^ and the second one usually arises from different surface contaminants, such as OH groups, CO_*x*_, weakly bonded oxygen, peroxide species, etc.^45,48^ Additionally, the oxygen signals from RuO_2_ can be hidden among that peak.^11^

### In-situ NAP-XPS characterization

The *in-situ* NAP-XPS study of the as-prepared Ru/CeO_2_ catalyst was conducted during various pretreatments and throughout the propane oxidation reaction under dry and humid conditions. It provided a more comprehensive understanding of the processes occurring on the catalyst surface under the in situ conditions, thereby assisting in elucidating the catalytic activity results presented above. The concentrations of O_2_, C_3_H_8_, and H_2_O gases used in the NAP-XPS measurements were 1.6 mbar, 0.2 mbar, and 1 mbar, respectively, which were only five times lower than those used in the catalytic tests. Unfortunately, performing the NAP-XPS studies at higher pressures to match the catalytic test conditions was not feasible due to the significant attenuation of the XPS signal.

**Figure 5:**
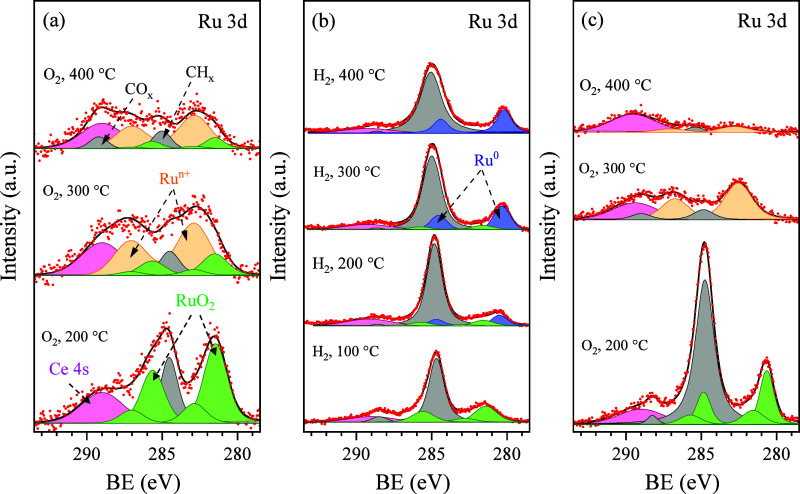
NAP-XPS Ru 3d spectra acquired from the as-prepared Ru/CeO_2_ catalyst during stepwise annealing at various temperatures in 1 mbar of O_2_ (a), 0.5 mbar of H_2_ (b), and during the subsequent reoxidation of the H_2_-annealed catalyst in 1 mbar of O_2_ (c).

Figure 5a shows Ru 3d spectra acquired from the as-prepared Ru/CeO_2_ catalyst upon stepwise annealing to 400 °C in 1 mbar of O_2_. The Ce 3d and O 1s spectra measured simultaneously with the Ru 3d spectra are presented in Figure S7 of the SI. Examining the evolution of the Ce 3d and O 1s spectra demonstrated that they closely resembled those obtained from the as-prepared Ru/CeO_2_ catalyst in UHV and remained unchanged during annealing in O_2_. The Ru 3d spectra revealed that the exposure of the sample to O_2_ at 200 °C results in Ru remaining on the surface in the Ru^4+^ oxidation state and only leads to decreased surface carbon contamination (bottom spectrum in Figure 4b). Raising the temperature to 300 °C led to the oxidation of the majority of Ru atoms to a higher oxidation state characterized by a Ru 3d doublet with the main peak at about 282.5 eV, which, according to the literature, may be assigned to highly oxidized Ru^n+^ (*n* > 4) species.^49^ It should be mentioned that some authors denied the existence of highly oxidized Ru species in the condensed form due to their low stability and explained the presence of a signal at such high BE as satellite or plasmon peaks of RuO_2_.^11,50^ However, our findings reveal that in a highly oxidizing environment at temperatures above 200 °C, small RuO_2_ NPs undergo oxidation to higher states, leading to the presence of Ru^n+^ ions on the surface, probably from the condensed RuO_3_ and RuO_4_ phases.

When the temperature was increased to 400 °C, an additional signal appeared at approximately 289 eV. This is likely attributable to the accumulation of carbonates on the surface or the partial oxidation of surface-bound carbon species. It can also be noticed that the total Ru signal in the Ru 3d spectrum decreased. The reduced signal of Ru obtained from the O_2_-exposed surface at 400 °C indicates that these oxides either begin to evaporate or diffuse into the ceria when the temperature exceeds 300 °C. Indeed, there are reports on Ru dissolution in CeO_2_, resulting in Ce_1–*x*_Ru_*x*_O_2_ formation in the subsurface layer of Ru/CeO_2_ catalyst.^10^ On the other hand, numerous studies report that Ru and CeO_2_ separate into two phases when calcined at temperatures above 400 °C.^51,52^ Furthermore, no evidence of Ru dissolution in ceria while annealing in oxygen was observed in the works of P. Liu et al.^15^ and Aitbekova et al.^7^ Instead, they reported the redispersion of Ru on the ceria surface. Additionally, in our previous work,^19^ a scanning electron microscopy EDS analysis of a 0.5 nm thick Ru layer deposited on a 30 nm thick CeO_2_ film before and after annealing at 500 °C in 1 mbar of O_2_ showed almost complete disappearance of Ru from the ceria surface. Considering that the depth of the EDS signal collection is approximately 1 μm (which is much higher than the total thickness of the studied Ru/CeO_2_ planar structures, about 30 nm), and the observation of the formation of highly oxidized Ru species on the ceria surface by NAP-XPS, we believe that the observed disappearance of Ru is more likely due to the evaporation of Ru in the form of volatile RuO_3_ or RuO_4_ species. We consider the temperature of 300 °C the highest possible calcination temperature that can be applied to the Ru/CeO_2_ catalyst without substantial loss of Ru from the surface. Notably, annealing the oxidized catalyst in an inert argon atmosphere (refer to Figure S8 in the SI) demonstrated that, in the absence of oxygen, the RuO_*x*_ species decompose on the surface of ceria to metallic Ru instead of undergoing evaporation.

Ru 3d spectra measured from the as-prepared Ru/CeO_2_ catalyst during stepwise heating in 0.5 mbar of H_2_ are presented in Figure 5b. It can be seen that the annealing at 100 °C only slightly increased the amount of surface carbon contamination and did not influence the oxidation state of Ru, which remain it in the Ru^4+^ form. It contradicted the H_2_-TPR data, showing the reduction of the as-prepared Ru/CeO_2_ catalyst at temperatures below 100 °C. This contradiction might come from the 2 orders of magnitude lower H_2_ partial pressure used in the NAP-XPS experiment, which usually impacts the kinetics and dynamics of the reduction process.^53^ Raising the temperature to 200 °C already demonstrated some reduction of RuO_2_ to metallic Ru, characterized by the Ru 3d_5/2_ line of the 3d doublet at about 280.2 eV.^11^ Eventually, at temperatures of 300 and 400 °C, the catalyst underwent substantial reduction and contained metallic Ru only. At 400 °C, the reduction of RuO_2_ was also accompanied by the significant reduction of ceria, as evidenced by the corresponding Ce 3d spectrum (refer to Figure S9a in the SI), indicating the appearance of Ce^3+^ ions on the surface due to the formation of numerous oxygen vacancies. The reduction of ceria can also be seen from the O 1s spectra measured simultaneously with the Ru 3d and Ce 3d spectra and presented in Figure S9b in the SI. The spectra showed a small shift of the main peak at 529.3 eV to higher BE with increasing temperature, which is typical behavior in the case of surface ceria reduction.^47^

The reoxidation of the reduced catalyst in O_2_ (Figure 5c and Figure S10 of the SI) revealed similar results as in the case of the as-prepared catalyst annealing in O_2_. However, in this case, the amount of surface carbon was much higher than on the as-prepared Ru/CeO_2_ catalyst. Additionally, the position of the Ru 3d_5/2_ line of 3d doublet after the reoxidation at 200 °C was about 281 eV, corresponding to anhydrous RuO_2_.^11^ Increasing the temperature to 300 °C resulted in the disappearance of all carbon and the oxidation of RuO_2_ to RuO_3_/RuO_4_, while at 400 °C, almost all Ru disappeared from the surface. It demonstrated that the reduction–oxidation cycles accelerate the evaporation of Ru from the ceria surface, which can be explained by the faster oxidation and evaporation kinetics of metallic Ru NPs compared to the hydrated RuO_2_ ones.

The most active reduced Ru/CeO_2_ catalyst was then studied under the propane oxidation reaction conditions. To comprehend the influence of water on the surface chemistry of the reduced Ru/CeO_2_ catalyst and elucidate its effect during propane oxidation, we performed measurements under both dry and humid conditions. The measurements under dry conditions were conducted in a 1.8 mbar O_2_/C_3_H_8_ (10:1) mixture, while humid conditions involved the additional introduction of 1 mbar of water vapor to create an O_2_/C_3_H_8_/H_2_O mixture with a total pressure of 2.8 mbar. Before the NAP-XPS measurements, the as-prepared catalyst underwent a 1 h annealing in 1 mbar of O_2_ at 300 °C and a half-hour annealing in 0.5 mbar of H_2_ at 400 °C in order to obtain the reduced Ru/CeO_2_ catalyst with minimized carbon contamination.

**Figure 6:**
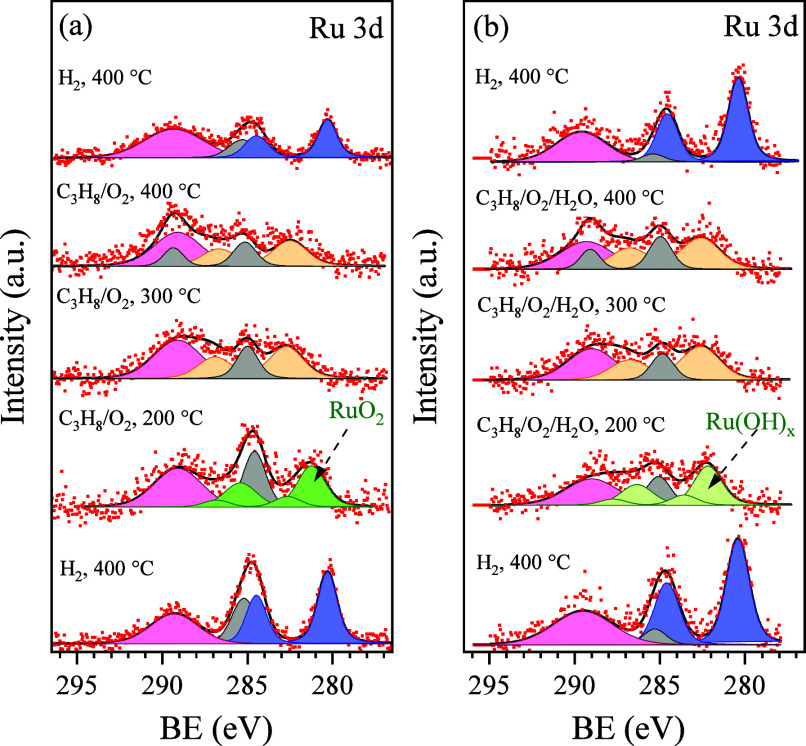
NAP-XPS Ru 3d spectra acquired from the reduced Ru/CeO_2_ catalyst during propane oxidation reaction at different temperatures in dry (a) and humid (b) conditions.

The Ru 3d NAP-XPS spectra obtained from the reduced Ru/CeO_2_ catalyst during H_2_ annealing and propane oxidation reactions under both dry and humid conditions are presented in Figure 6. The corresponding Ce 3d and O 1s spectra measured simultaneously with the Ru 3d spectra are shown in Figure S11 in the SI. Under dry conditions (Figure 6a), the metallic Ru NPs formed during H_2_ reduction reoxidized into RuO_2_ (Ru 3d doublet with the main peak at about 281 eV) upon exposure to the O_2_/C_3_H_8_ mixture at 200 °C — similar to the reduced catalyst’s response to oxygen alone. The small amount of propane in the gas atmosphere did not influence the catalyst surface chemistry at higher temperatures either. Similarly, as in the case of O_2_ only, elevating the sample temperature to 300 and 400 °C resulted in the farther oxidation of most Ru^4+^ and the appearance of the Ru^n+^ ions on the surface. Conversely, in humid conditions, the catalyst exhibited a different behavior. As shown in Figure 6b, introducing water vapor into the exposure atmosphere at 200 °C resulted in the appearance of Ru 3d states similar to those observed under dry conditions but shifted by approximately 1 eV to higher binding energy (around 282 eV). Since these peaks were still about 0.5 eV lower than the Ru^n+^ doublet, we attribute them to Ru(OH)_*x*_ species, which are reported to appear at approximately 282 eV.^11^ At 300 and 400 °C, the Ru 3d spectrum collected in the humid atmosphere resemble that obtained under dry conditions. In the dry experiment, postannealing of the samples in H_2_ revealed approximately half the amount of ruthenium compared to the prereaction state. In contrast, the presence of water in the exposure atmosphere significantly mitigated the loss of Ru, with the intensity of Ru peaks dropping only slightly (by 10–15%). This stabilization effect might be attributed to OH groups, which potentially trap Ru–O_x_ complexes during the oxidative redispersion of Ru on the ceria surface.^54^

The results presented above demonstrate the oxidation and evaporation of Ru from the ceria surface upon heating in the highly oxidizing environment of propane oxidation. We believe these phenomena can explain the observed catalyst activity behavior during propane oxidation tests in the catalytic reactor. Indeed, according to the NAP-XPS results, after the calcination of the powder-like Ru/CeO_2_ catalyst in oxygen at 500 °C, all Ru NPs present inside had to oxidize, partially evaporate, and most probably deposit as a thin RuO_*x*_ (*x* > 2) layer on the surface of ceria grains. This process likely occurs concurrently with the widely reported oxidative redispersion of Ru and may, in fact, facilitate this redispersion. The annealing of such an oxidized catalyst in the inert atmosphere showed that the RuO_*x*_ layer is unstable and tends to decompose to RuO_2_ at temperatures below 200 °C. Thus, it can be concluded that at lower temperatures before introducing the propane-oxygen mixture to the reactor, the catalysts most probably contained Ru^4+^ ions finely dispersed on the surface of stoichiometric ceria. Such a surface appears to exhibit relatively low catalytic activity for propane oxidation at 200 °C, both under dry and humid conditions. As the temperature in the reactor increased to 300 °C or more, the Ru^4+^ ions should have been oxidized to the Ru^n+^ ones, and such a surface demonstrates relatively high catalytic activity toward the oxidation of propane. The inertness of the oxidized catalyst to water is likely associated with the generally low reactivity of fully oxidized ceria and ruthenia toward water adsorption.^55,56^ It also appears that the process of RuO_*x*_ evaporation from the open catalyst surface that occurs when the catalyst is maintained in the oxidizing environment at such high temperatures may lead to the gradual washing out of RuO_*x*_ from the catalytic reactor. As mentioned before, this process can explain the decreased catalyst activity on the decreasing temperature ramp shown in Figure 3a. However, other processes, such as sintering or coking, may also contribute to this phenomenon. Nevertheless, the NAP-XPS study did not reveal coke formation on the surface during the reaction, and STEM analysis showed a relatively unchanged size of ceria crystallites after O_2_ calcination.

The reduced Ru/CeO_2_ catalyst exhibited significantly higher catalytic activity at low temperatures under both dry and humid conditions. We attribute this to the presence of Ru in the catalyst in the form of RuO_2_ and Ru(OH)_*x*_ nanoparticles. We hypothesize that at lower temperatures, metallic Ru NPs undergo oxidation but do not yet redisperse across the surface of ceria, as this process typically occurs at around 230–250 °C.^7^ These oxidized Ru NPs may exhibit higher catalytic activity than the highly dispersed Ru^4+^ ions in the calcined catalyst. This aligns with findings in the literature highlighting Ru or RuO_2_ NPs as active phases in Ru/CeO_2_.^12,57^ Indeed, the TEM measurements showed that reducing Ru/CeO_2_ results in the formation of small metallic Ru NPs on the surface of ceria grains, which oxidize to RuO_2_ in ambient air at RT. Thus, it is reasonable to expect that the tiny metallic Ru NPs will oxidize to RuO_2_ in the catalytic reactor immediately after exposure to the reaction mixture. Consequently, the reaction at low temperatures proceeds on a catalyst surface containing RuO_2_ NPs, as also evidenced by the NAP-XPS results. Our catalytic data at 200 °C show that such catalyst exhibited several-fold higher activity than the oxidized catalyst containing atomically dispersed Ru^4+^. However, as the temperature increased to 300 °C, the RuO_2_ NPs began to oxidize to RuO_*x*_ through the above-mentioned processes of redispersion and evaporation, transforming the reduced catalyst into the oxidized one with atomically dispersed Ru. This transformation is reflected in the decline of catalyst activity at 300 and 400 °C until it aligns with the oxidized catalyst activity at 500 °C and already acts as the oxidized catalyst during the decreasing temperature ramp.

It should also be mentioned that the prereduction of CeO_2_ may also contribute to the increased activity of the reduced Ru/CeO_2_ catalyst, as it typically influences the oxygen-transport ability of ceria and creates different surface defects that may adsorb aforementioned weakly bonded oxygen species. These phenomena are essential for chemical reactions proceeding via the Mars-van-Krevelen mechanism.^31,58^ On the other hand, the Ce 3d and O 1s spectra collected during propane oxidation at 200 °C (Figure S11 of the SI) seem identical to the spectra obtained from the oxidized catalyst in O_2_ (Figure S7 of SI), evidencing the full reoxidation of the ceria surface. Therefore, in our opinion, the observed difference in the activities of the reduced and oxidized catalysts under dry conditions most probably originates from the different structures of RuO_2_ species. The interaction of water with the RuO_2_ NPs of the reduced catalyst and formation of Ru(OH)_*x*_ under humid conditions, which have a boosting effect on catalyst activity, suggest that the activation of propane on Ru(OH)_*x*_ NPs is more efficient or easier than on the RuO_2_ ones. Also, the mechanism of propane oxidation probably involves the interaction of propane with OH groups, supplied by Ru(OH)_*x*_.

## Conclusions

In this work, we performed a comprehensive study of the stability and activity of differently pretreated CeO_2_-supported Ru catalysts during the oxidation of propane under dry and humid conditions, which is essential for understanding the catalyst behavior under the industrially relevant conditions of VOC oxidation. The results demonstrated that the catalyst calcined in O_2_ at 500 °C starts to oxidize propane already at 200 °C and reaches a high conversion rate at temperatures above 400 °C. The presence of humidity in the reaction mixture had a negligible effect on the catalyst activity across the entire studied temperature range (200–500 °C). The prereduction in H_2_ of the oxidized Ru/CeO_2_ catalyst improved the catalyst activity by a one-order of magnitude; however, this improvement diminished with the increase in reaction temperature. Adding water to the reaction mixture further improved low-temperature activity; however, at 300–400 °C, this activity gradually decreased with time, and at 500 °C, the activity became identical to the activity of the oxidized catalyst. The *in-situ* NAP-XPS investigation of the surface chemistry of the Ru/CeO_2_ catalyst during oxidizing and reducing pretreatments, as well as propane oxidation reactions under both dry and humid conditions, clearly revealed that small Ru metallic nanoparticles dispersed on the ceria surface oxidize in oxygen-containing environments at temperatures below 200 °C to form RuO_2_, and at higher temperatures to RuO_*x*_ (*x* > 2) species, which tend to evaporate slowly from the surface. This finding may contribute to the discussion on the mechanism of Ru redispersion, a phenomenon widely reported in the literature. It is demonstrated that the oxidized RuO_2_ and Ru(OH)_*x*_ NPs exhibit higher catalytic activity than the highly dispersed Ru^4+^ ions present in the calcined catalyst. It is also revealed that at typical reaction temperatures for VOC oxidation over Ru/CeO_2_ catalysts (above 300 °C), Ru exists in the oxidation state of Ru^n+^ (*n* > 4) rather than the commonly believed Ru^4+^ state.

## Supporting Information Available

Temperature ramping used for the catalyst activity measurements; XRD diffractograms; STEM/EDS analyses of the as-prepared and oxidized Ru-CeO_2_ catalysts; CO_2_ production rates during the oxidation of propane at a higher concentration (0.24 vol %); dependence of the CO_2_ production rates on the partial pressure of propane; Arrhenius plots used for the calculation of the reaction activation energies; *ex-situ* XPS Ce 3d and O 1s spectra acquired from the as-prepared Ru/CeO_2_ catalyst and the catalysts that passed the catalytic tests; NAP-XPS Ce 3d and O 1s spectra of the as-prepared Ru/CeO_2_ catalyst during the stepwise annealing in 1 mbar of O_2_; NAP-XPS Ru 3d spectra of the oxidized Ru/CeO_2_ catalyst during the stepwise annealing in 1 mbar of Ar; NAP-XPS Ce 3d and O 1s spectra of the as-prepared Ru/CeO_2_ catalyst during the stepwise annealing in 0.5 mbar of H_2_; NAP-XPS Ce 3d and O 1s spectra of the reduced Ru-CeO_2_ catalyst during the reoxidation in 1 mbar of O_2_; NAP-XPS Ce 3d and O 1s spectra of the reduced Ru/CeO_2_ catalyst during propane oxidation in dry and humid conditions; comparison of the catalytic activity of Ru/CeO_2_ catalyst obtained in this work with values reported in the literature (PDF)
