# Supplementary material for: Unraveling the Effects of Reducing and Oxidizing Pretreatments and Humidity on the Surface Chemistry of the Ru/CeO2 Catalyst during Propane Oxidation
Source: J Phys Chem C Nanomater Interfaces. 2025 Jan 14;129(3):1746–57. doi: 10.1021/acs.jpcc.4c08033 (PMC11770751; doi:10.1021/acs.jpcc.4c08033)
Supplement: Supplementary file 1 — jp4c08033_si_001.pdf [file jp4c08033_si_001.pdf]

Supporting Information

Unraveling the Effects of Reducing and Oxidizing Pretreatments and Humidity on the Surface Chemistry of Ru/CeO_2 Catalyst during Propane Oxidation

Thu Ngan Dinhová[1], Oleksii Bezkrovnyi[2], Lesia Piliai[1], Ivan Khalakhan[1], Samiran Chakraborty[1], Maciej Ptak[2], Piotr Kraszkiewicz[2], Mykhailo Vaidulych[3], Michal Mazur[4], Štefan Vajda[3], Leszek Kepinski[2], Michael Vorochta[1*], Iva Matolínová[1]*

[1]Department of Surface and Plasma Science, Faculty of Mathematics and Physics, Charles University, V Holešovičkách 2, Prague 180 00, Czechia

[2]W. Trzebiatowski Institute of Low Temperature and Structure Research, Polish Academy of Sciences, Okólna 2,Wroclaw 50-422, Poland

[3]Department of Nanocatalysis, J. Heyrovský Institute of Physical Chemistry, Czech Academy of Sciences, Dolejškova 2155/3, Prague 182 23, Czechia

[4]Department of Physical and Macromolecular Chemistry, Faculty of Science, Charles University, Hlavova 8, Prague 128 43, Czechia

[*] Corresponding authors: o.bezkrovnyi@intibs.pl; vorokhtm@mbox.troja.mff.cuni.cz

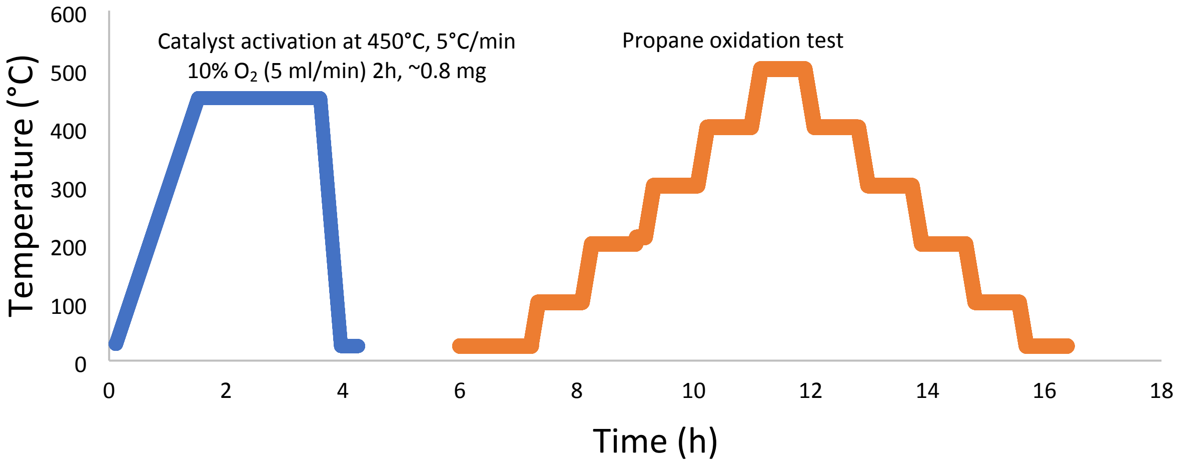

Fig. S1. Temperature ramping used in the Ru-CeO_2 catalyst propane oxidation activity measurements.

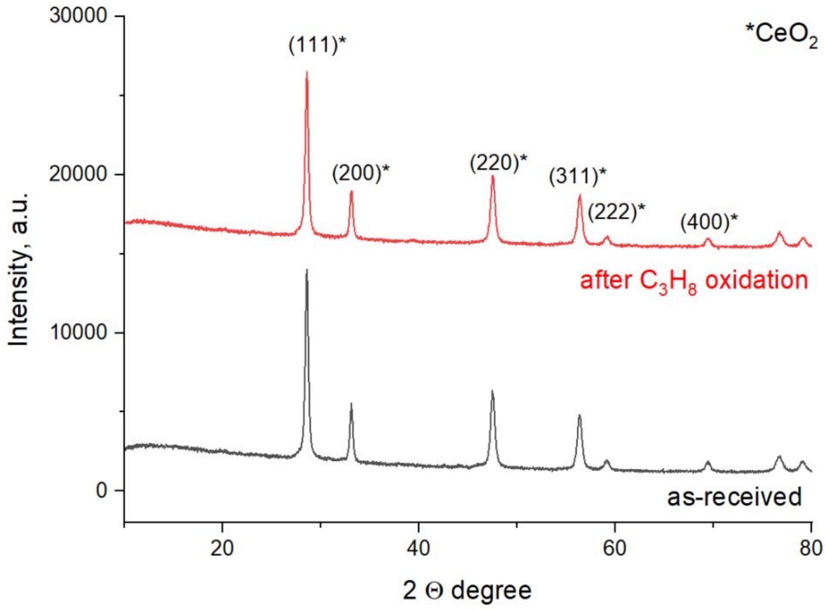

Fig. S2. Ex-situ XRD diffractograms of the as-prepared Ru-CeO_2 catalyst and the Ru-CeO_2 catalyst after the propane oxidation activity measurements.

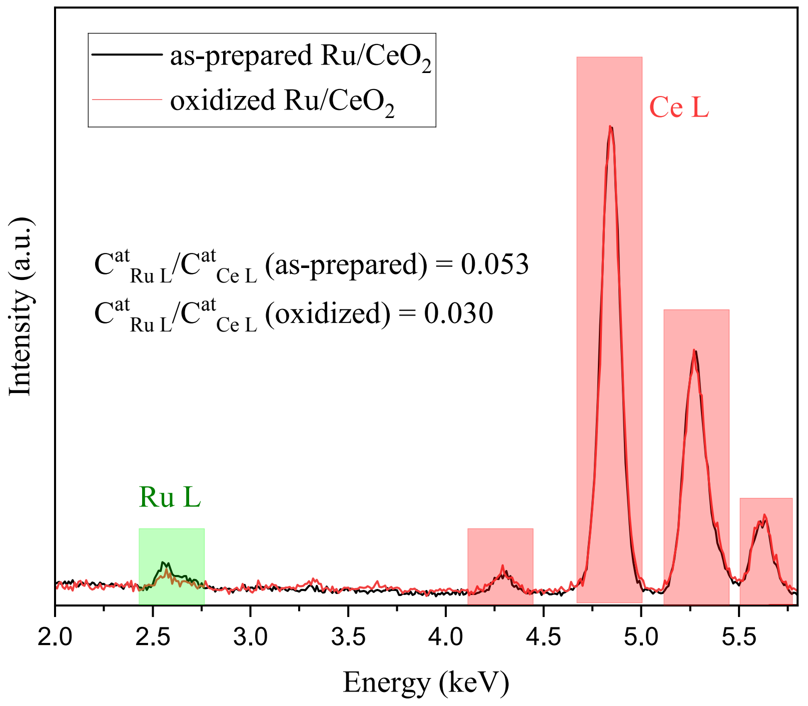

Fig. S3. Comparison of the integrated STEM/EDS signal for the as-prepared and oxidized Ru-CeO_2 catalysts.

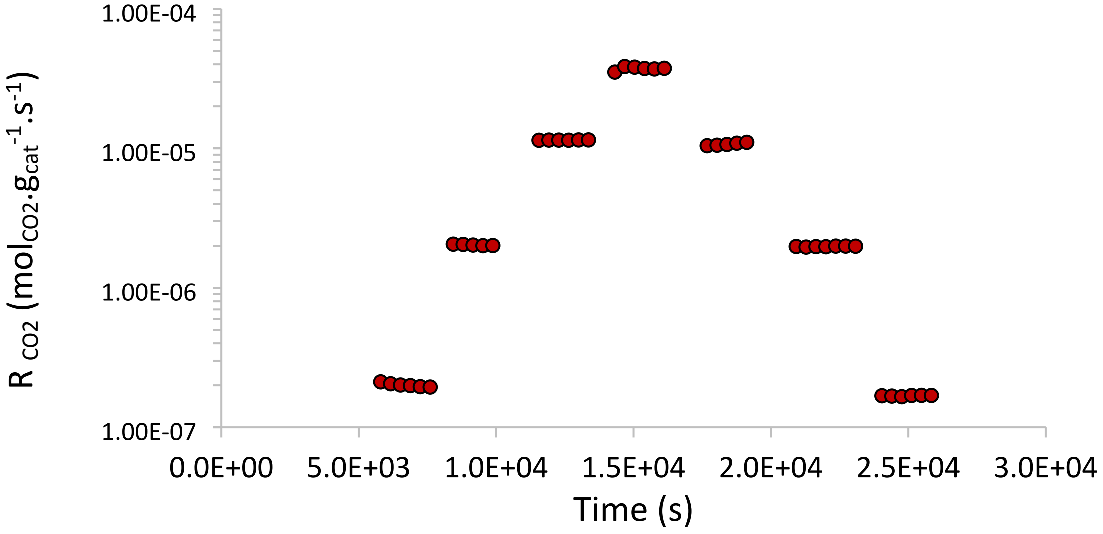

Fig. S4. Time-resolved CO_2 production rates during propane oxidation on the oxidized Ru/CeO_2 catalyst under dry (0.24 vol % C_3H_8–2.4 vol % O_2–97.36 vol % He).

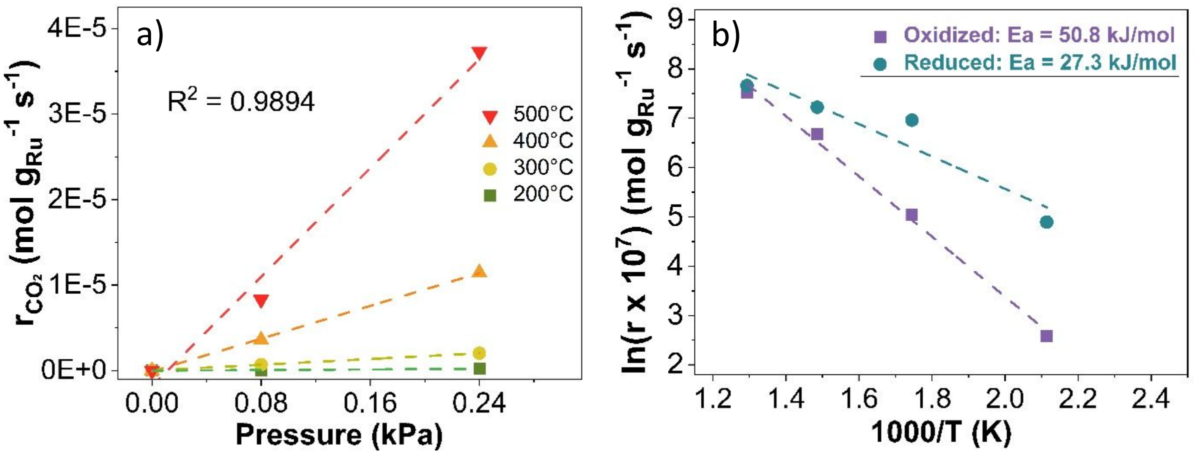

Fig. S5. (a) Dependence of the CO_2 production rates during the oxidation of propane in dry conditions over the oxidized Ru/CeO_2 catalyst on the partial pressure of propane in the flow of gasses entering the catalytic reactor; (b) The Arrhenius plots used to calculate the reaction activation energy (E_a) for the oxidized and reduced Ru/CeO_2 catalysts.

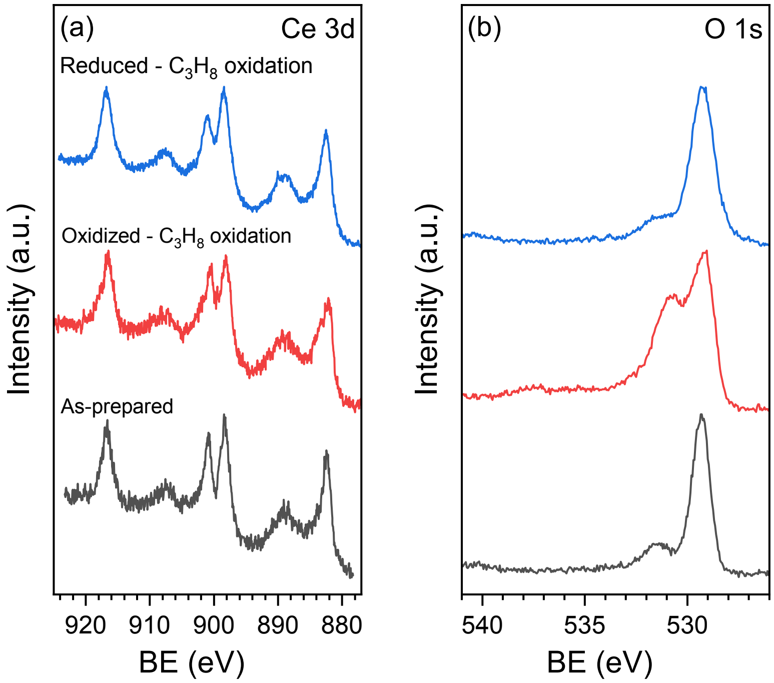

Fig. S6. *Ex-situ* UHV-XPS Ce 3d (a) and O 1s (b) spectra acquired from the as-prepared Ru/CeO_2 catalyst, and the oxidized and reduced Ru/CeO_2 catalysts that passed the catalytic test in the catalytic reactor for propane oxidation.

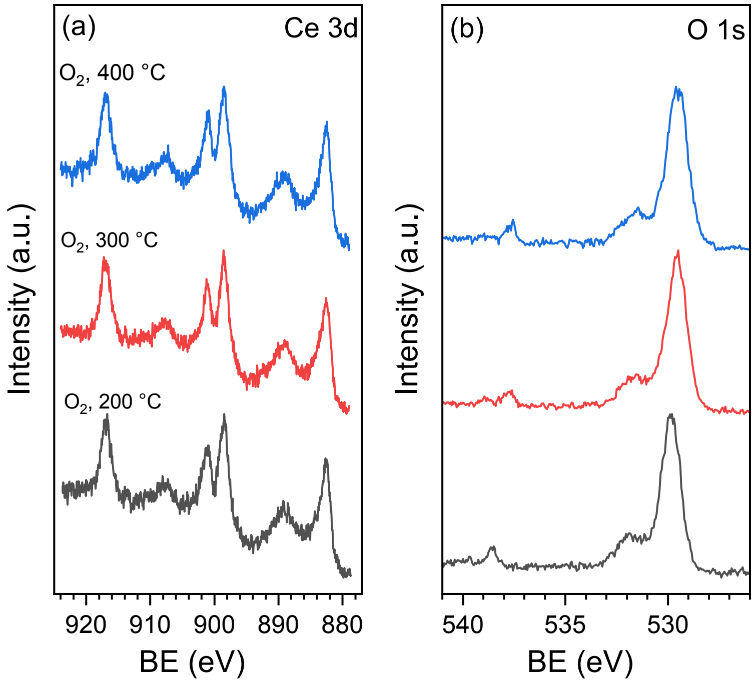

Fig. S7. NAP-XPS Ce 3d (a) and O 1s (b) spectra acquired from the as-prepared Ru/CeO_2 catalyst during the stepwise increase of the sample temperature in 1 mbar of O_2.

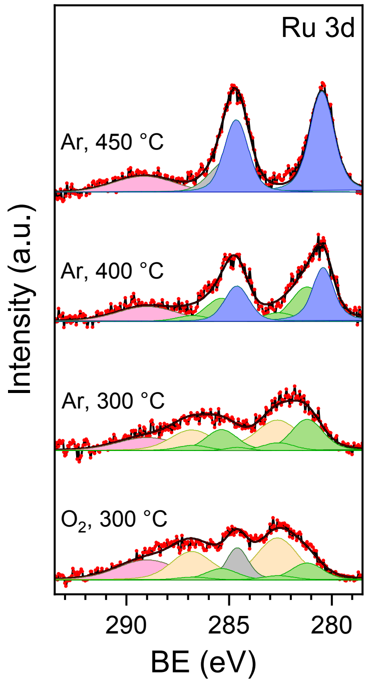

Fig. S8. NAP-XPS Ru 3d spectra acquired from the Ru/CeO_2 catalyst in O_2 at 300 °C and upon the subsequent stepwise increase of the sample temperature in 1 mbar of Ar.

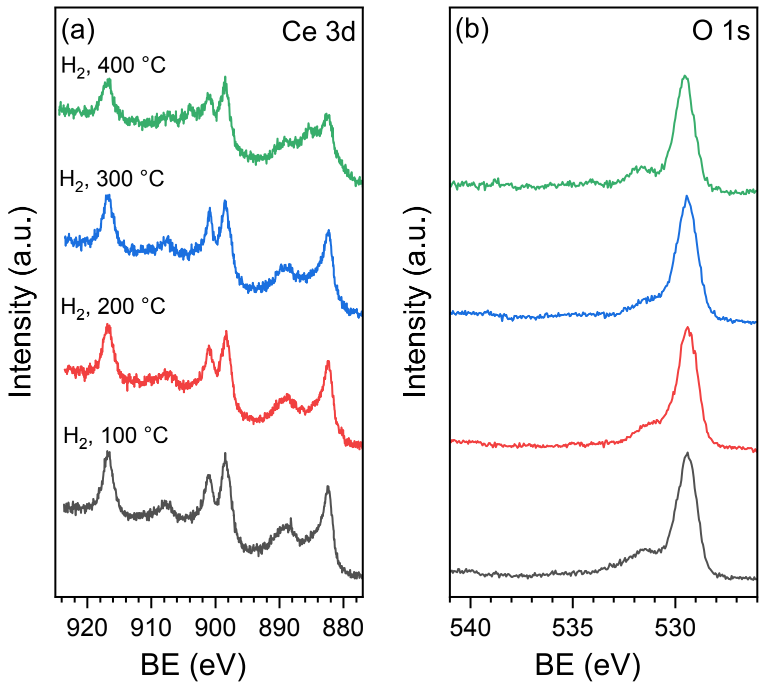

Fig. S9. NAP-XPS Ce 3d (a) and O 1s (b) spectra acquired from the as-prepared Ru/CeO_2 catalyst during the stepwise increase of the sample temperature in 0.5 mbar of H_2.

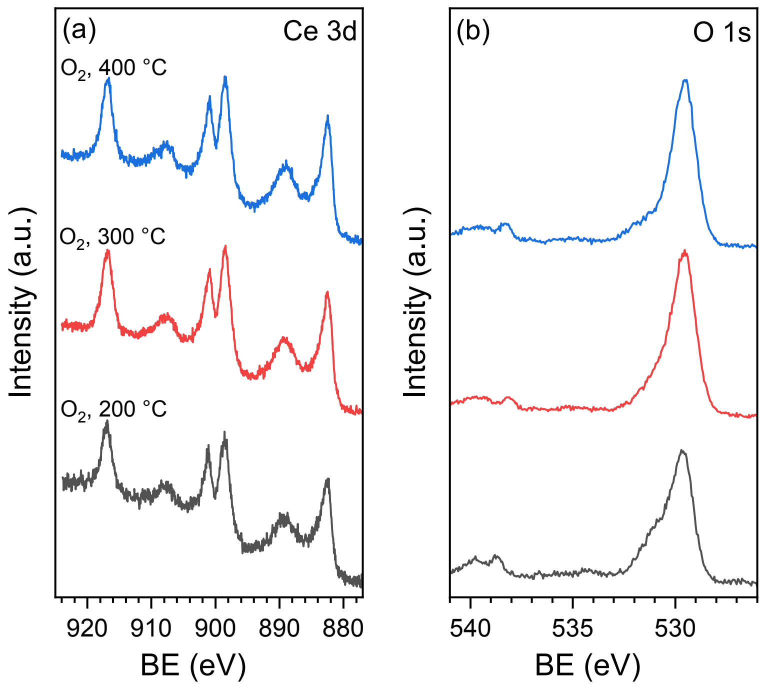

Fig. S10. NAP-XPS Ce 3d (a) and O 1s (b) spectra acquired during the re-oxidation of the H_2-annealed catalyst in 1 mbar of O_2 (c).

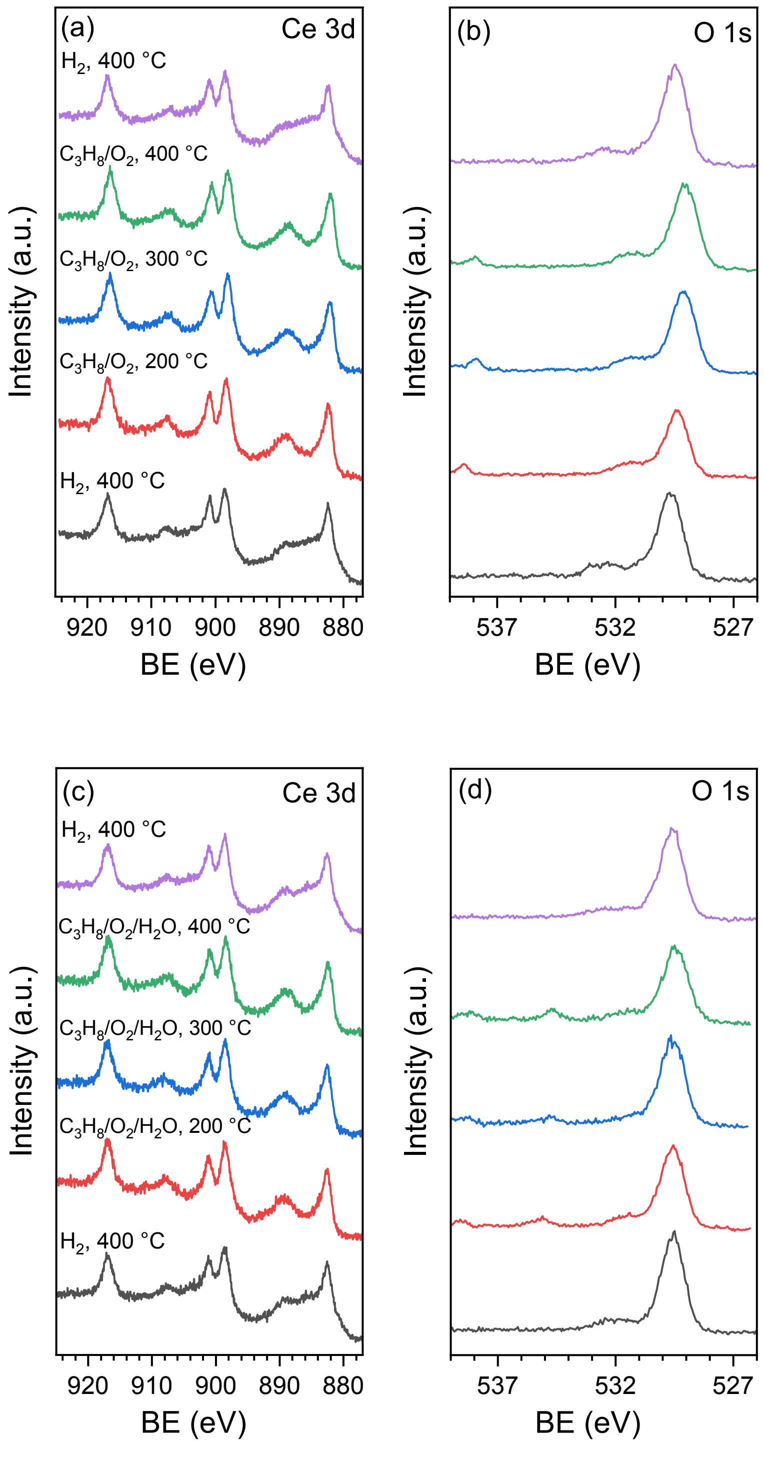

Fig. S11. NAP-XPS Ce 3d and O 1s spectra acquired from the reduced Ru/CeO_2 catalyst during propane oxidation in dry (a, b) and humid (c, d) conditions.

Table S1. Comparison of the catalytic activity (conversion of propane) of the oxidized and reduced Ru/CeO_2 catalyst with values reported in the literature.

Catalyst	Temperature K	The feed gas	R_{C3H8}*10^6 mol.g $_{Ru}$ $^{-1}$.s^{-1}	Reference
Oxidized Ru/CeO_2	473	0.08 vol % C_3H_8–0.8 vol % O_2–99.12 vol % He	1.41	This work
Oxidized Ru/CeO_2		0.08 vol % C_3H_8–0.8 vol % O_2–0.04% H_2O–99.08 vol % He	1.52	This work
Reduced Ru/CeO_2		0.08 vol % C_3H_8–0.8 vol % O_2–99.12 vol % He	13.7	This work
Reduced Ru/CeO_2		0.08 vol % C_3H_8–0.8 vol % O_2–0.04% H_2O–99.08 vol % He	21.5	This work
Oxidized Ru/CeO_2	573	0.08 vol % C_3H_8–0.8 vol % O_2–99.12 vol % He	15.5	This work
Oxidized Ru/CeO_2		0.08 vol % C_3H_8–0.8 vol % O_2–0.04% H_2O–99.08 vol % He	14.5	This work
Reduced Ru/CeO_2		0.08 vol % C_3H_8–0.8 vol % O_2–99.12 vol % He	105.6	This work
Reduced Ru/CeO_2		0.08 vol % C_3H_8–0.8 vol % O_2–0.04% H_2O–99.08 vol % He	110.2	This work
Oxidized Ru/CeO_2	673	0.08 vol % C_3H_8–0.8 vol % O_2–99.12 vol % He	79.7	This work
Oxidized Ru/CeO_2		0.08 vol % C_3H_8–0.8 vol % O_2–0.04% H_2O–99.08 vol % He	90	This work
Reduced Ru/CeO_2		0.08 vol % C_3H_8–0.8 vol % O_2–99.12 vol % He	136.7	This work
Reduced Ru/CeO_2		0.08 vol % C_3H_8–0.8 vol % O_2–0.04% H_2O–99.08 vol % He	126.2	This work
Oxidized Ru/CeO_2	773	0.08 vol % C_3H_8–0.8 vol % O_2–99.12 vol % He	184.3	This work
Oxidized Ru/CeO		0.08 vol % C_3H_8–0.8 vol % O_2–0.04% H_2O–99.08 vol % He	169.1	This work
Reduced Ru/CeO_2		0.08 vol % C_3H_8–0.8 vol % O_2–99.12 vol % He	212.2	This work
Reduced Ru/CeO_2		0.08 vol % C_3H_8–0.8 vol % O_2–0.04% H_2O–99.08 vol % He	190.9	This work
Ru/CeO_2-1	473	0.2 vol.% C_3H_8, 2 vol.% O_2, 97.8 vol.% Ar	35.2	[1]
Ru/CeO_2-2	473	0.2 vol.% C_3H_8, 2 vol.% O_2, 97.8 vol.% N_2	23.9	[1]
Ru/CeO_2-3	473	0.2 vol.% C_3H_8, 2 vol.% O_2, 97.8 vol.% N_2	15.8	[1]
Ru/Al_2O3-1	473	0.2 vol.% C_3H_8, 2 vol.% O_2, 97.8 vol.% N_2	8.46	[1]
Ru/Al_2O3-2	473	0.2 vol.% C_3H_8, 2 vol.% O_2, 97.8 vol.% N_2	6.18	[1]
Ru/Al_2O3-3	473	0.2 vol.% C_3H_8, 2 vol.% O_2, 97.8 vol.% N_2	5.21	[1]
Ru/CeO_2	473	0.2 vol.% C_3H_8, 2 vol.%	25.2	[2]

		O_2, 97.8 vol.% Ar		
Ru/Co_3O_4	473	0.2 vol.% C_3H_8, 2 vol.% O_2, 97.8 vol.% Ar	18.9	[2]
Ru/CeO_2-R	428	0.2 vol.% C_3H_8, 2 vol.% O_2, 97.8 vol.% Ar	21.26	[3]
Ru/CeO_2-R	428	0.2 vol.% C_3H_8, 2 vol.% O_2, 97.8 vol.% Ar	7.81	[3]
Ru/CeO_2-R	428	0.2 vol.% C_3H_8, 2 vol.% O_2, 97.8 vol.% Ar	1.25	[3]

References

[1] Z. Hu, Z. Wang, Y. Guo, L. Wang, Y. Guo, J. Zhang, W. Zhan, Total Oxidation of Propane over a Ru/CeO2 Catalyst at Low Temperature, Environ. Sci. Technol. 52 (2018) 9531–9541.

[2] A. Wang, J. Ding, M. Li, P. Song, Z. Zhao, Y. Guo, Y. Guo, L. Wang, Q. Dai, W. Zhan, Robust Ru/Ce@Co Catalyst with an Optimized Support Structure for Propane Oxidation, Environ. Sci. Technol. 58 (2024) 12742–12753.

[3] Z. Wang, Z. Huang, J.T. Brosnahan, S. Zhang, Y. Guo, Y. Guo, L. Wang, Y. Wang, W. Zhan, Ru/CeO2 Catalyst with Optimized CeO2 Support Morphology and Surface Facets for Propane Combustion, Environ. Sci. Technol. 53 (2019) 5349–5358.
